# The miR-941/FOXN4/TGF-β feedback loop induces N2 polarization of neutrophils and enhances tumor progression of lung adenocarcinoma

**DOI:** 10.3389/fimmu.2025.1561081

**Published:** 2025-04-25

**Authors:** Xiaojing Zhang, Xitong Huang, Xianying Zhang, Lichang Lai, Baoyi Zhu, Peibin Lin, Zhanfang Kang, Dazhong Yin, Dongbo Tian, Zisheng Chen, Jun Gao

**Affiliations:** ^1^ Department of Respiratory and Critical Care Medicine, The Affiliated Qingyuan Hospital (Qingyuan People’s Hospital), Guangzhou Medical University, Qingyuan, China; ^2^ Department of Traditional Chinese Medicine, China Pharmaceutical University, Nanjing, China

**Keywords:** lung adenocarcinoma (LUAD), single-cell RNA sequencing (scRNA-seq), tumor-associated neutrophils (TANs), miR-941, Foxn4, TGF-b

## Abstract

**Background:**

Lung adenocarcinoma (LUAD) is a major subtype of lung cancer and one of the deadliest cancers in humans. Dysregulation of miRNA activity in tumor-associated neutrophils (TANs) in the tumor microenvironment plays an important role in the occurrence and development of LUAD.

**Method:**

In this study, the miReact algorithm was used to analyze the single-cell RNA sequencing data of LUAD samples to reveal the miRNA profile characteristics of TANs in LUAD patients. The function of miR-941 was investigated *in vivo* and *in vitro*. The target gene and underlying signaling pathway of miR-941 were predicted and validated with qPCR, luciferase assay, WB and ELISA assay.

**Results:**

The results indicated the crucial role of TANs, especially N2-TANs in LUAD and miR-941 activity was significantly upregulated in TANs of LUAD patients. MiR-941 overexpression promoted the proliferation, invasion, migration and anti-apoptosis of A549 and H1299. *In vivo* xenograft mouse model confirmed that miR-941 overexpression enhanced the growth of tumors formed by H1299 cells. Bioinformatics analysis showed that miR-941 targeted the tumor suppressor gene FOXN4, and we confirmed that FOXN4 overexpression could counteract the malignant effects of miR-941. In addition, miR-941 may drive LUAD progression through the FOXN4/TGF-β feedback signaling loop and participate in the N2-TAN polarization.

**Conclusion:**

In summary, these findings reveal the key role of N2-TANs and the miR-941/FOXN4/TGF-β signaling loop in LUAD progression and provide potential therapeutic targets for future interventions.

## Introduction

Lung cancer, a pervasive disease, is the primary cause of cancer-related mortality worldwide (1, 2). Lung cancer is classified as small-cell lung carcinoma (roughly 20% of cases) or non-small cell lung carcinoma (NSCLC, approximately 80% of cases) (3, 4). Lung adenocarcinoma (LUAD) is the predominant subtype of NSCLC, accounting for approximately 40% of all malignant lung tumors (5). The etiology of LUAD is linked to genetic mutations and environmental factors, including cigarette smoking and exposure to radiation and toxic substances (6–9). Despite advancements in diagnosis and treatment, the prognosis remains disconcertingly low, with just 15% of LUAD patients surviving beyond five years (10–14). This situation underscores the need to unravel the intricate mechanisms governing LUAD progression and identify new strategies to diagnose and treat the disease.

In past decades, the central role of the tumor microenvironment (TME) in the initiation and progression of lung carcinoma has been recognized (15–17). Myeloid cells play important roles in both innate immunity (18, 19) and tumorigenesis (20, 21). Tumor-infiltrating myeloid cells comprise monocytes, macrophages, dendritic cells, and neutrophils and have emerged as key regulators of cancer growth (22, 23). Neutrophils, extensively studied in autoimmune and infectious diseases (24–26), have gained prominence in cancer progression (27–29). Many studies have described neutrophils as key drivers of cancer progression and tumor promotion due to neutrophils’ participation in various tumor-promoting functions, including proliferation, aggressiveness, and dissemination (30–34). TANs show a strong association with lung cancer progression (35). Nevertheless, the molecular mechanism of tumor-associated neutrophils (TANs) in LUAD remains largely unclear.

Recent studies have revealed the heterogeneity of TANs in the TME, demonstrating their potential to polarize into subpopulations with distinct functions. Fridlender et al. first proposed the N1/N2 functional classification of TANs in 2009 (36). Similar to the M1/M2 macrophage classification, N1 neutrophils exhibit proinflammatory and antitumor properties, whereas N2 neutrophils possess anti-inflammatory and protumor functions (37). The mechanism of polarization of N1/N2 TANs in LUAD is unclear. Single-cell RNA sequencing (scRNA-seq) provides a powerful tool for investigating cellular heterogeneity within the immune microenvironment of lung cancer (38). This technology enables researchers to reveal the characteristics of individual cells at the molecular level, thereby facilitating a deeper understanding of the complexity of TAN heterogeneity (39). Analyzing the gene expression profiles of individual neutrophils using scRNA-seq can help to elucidate the dynamic changes and regulatory mechanisms of N1/N2 neutrophils in the lung cancer microenvironment.

MicroRNAs (miRNAs) are an abundant class of small, noncoding RNAs, approximately 19–25 nucleotides long. They modulate the expression of target genes by interacting with the 3’ untranslated regions (3’-UTRs) of target mRNAs and play an essential role in the biological and pathological processes of cancer (40, 41). Many studies have indicated that miRNAs can modulate LUAD tumor initiation and progression (16, 42–44). For instance, let-7b-5p was reported to regulate LUAD growth by PI3K/AKT signaling pathway (45). Neutrophils can secrete miRNA through vesicular structures, such as exosomes (46). miRNA secreted by neutrophils, such as miR-4466, can affect the proliferation, migration, invasion, and apoptosis of lung cancer cells (47). However, the involvement of miRNAs in the intricate interplay between N1/N2 TANs and LUAD progression remains unclear. In single-cell sequencing (scRNA-seq), direct measurement of miRNA activity often presents challenges. The miReact algorithm was designed to infer miRNA activity based on single-cell mRNA expression data. This algorithm leverages the negative regulatory relationship between miRNAs and their target genes to estimate miRNA activity levels by analyzing gene expression data from individual cells (48). Using this method, researchers can deduce miRNA activity from mRNA expression data without the need for direct measurement of miRNA expression. This provides a novel way of investigating the role of miRNAs in disease development.

In this study, we aimed to investigate the role of miRNA secreted from TANS in the pathology progression of LUAD. The results revealed TNA2-derived miR-941 as a central hub gene, and functional assays utilizing miR-941 mimics were conducted *in vitro* and *in vivo* to examine its oncogenesis role. In addition, the targets of miR-941 and the underlying signaling pathway were predicted and validated, the results of which suggested an miR-941/FOXN4/TGF-β feedback signaling loop in LUAD. These findings point to the existence of a novel cross-talk molecular axis for communication between TANs and cancer cells.

## Materials and methods

### Data collection

LUAD scRNA-seq datasets (GSE123902, GSE131907, and GSE136246) were obtained from the GEO database. The GSE123902 dataset contained four nontumor-involved lung samples and eight primary LUAD samples. The GSE131907 dataset contained 11 distant normal lung and four lung tumor tissue samples from patients with advanced-stage LUAD. The GSE136246 dataset contained LUAD samples from 12 patients.

### ScRNA-seq analysis

After obtaining a gene count matrix, quality control and dimensionality reduction clustering analyses were performed using Seurat (Version 3). High-quality single cells that passed the filtering step were first normalized using the NormalizeData function, applying log normalization to generate a standardized gene expression matrix. The FindVariableFeatures function was then employed to identify genes with significant expression variability among cells, with the default selection of the top 2,000 highly variable genes used for subsequent analyses. To address batch effects across the three GEO datasets, we applied the ​FindIntegrationAnchors function in Seurat (v3) with the following parameters: ​(1) dims = 1:30 (using the first 30 principal components to capture major sources of variation), ​(2) k.anchor = 5 (default, balancing sensitivity and computational efficiency for anchor identification), and ​(3) k.filter = 200 (filtering anchors to retain the top 200 mutual nearest neighbors). These parameters were selected to prioritize robust identification of shared biological states (e.g., myeloid subsets) while minimizing technical variability between datasets. Dimensionality reduction was conducted using the RunPCA function to determine the number of principal components for subsequent clustering. Cell clustering was performed using the FindNeighbors and FindClusters functions (with a default resolution of 1). After obtaining the gene count matrix, quality control was performed to exclude cells with <200 or >5,000 detected genes, mitochondrial gene content >10%, or <1,000 UMIs. High-quality cells were normalized using the LogNormalize method (scale factor = 10,000). Highly variable genes (top 2,000) were selected for PCA-based dimensionality reduction (20 components). The uniform manifold approximation and projection (UMAP) was utilized for visualizing single-cell populations.

### Identification of differentially expressed genes

Seurat’s FindMarkers function was used to perform differential gene expression analysis in different groups (using the bimod method for comparison), screen for DEGs in different clusters, and define DEGs with a fold change of ≥1.5 (corrected *p*-value ≤ 0.05) and at least a cell expression proportion greater than 0.1 (i.e., 10%) in at least one group.

### Determination of miRNA activity

The miRNA activity in the scRNA-seq data was determined using the miReact algorithm. Previous studies have demonstrated that motif enrichment analysis can estimate miRNA activity from scRNA-seq data (48). For each sample or cell, we ranked genes according to their fold change values. We then calculated miRNA activity based on the probabilistic outputs of motif enrichment tools, specifically Regmex software and Sylamer. We input the ordered 3’-UTRs into Sylamer, where motif probabilities were precalculated (48).

### Cell culture

Two human LUAD cell lines, A549 and H1299, were obtained from the American Type Culture Collection (ATCC, USA) and cultured at 37°C in a humidified atmosphere (95% air, 5% CO_2_). The A549 and H1299 cells were kept in Dulbecco’s Modified Eagle’s medium (Gibco, Germany) supplemented with 10% FCS (Germany), 2 mM l-glutamine, 10^5^ U/L penicillin, and 100 mg/L streptomycin.

### Scratch healing assay

In the investigation for miR-941 function, A549 and H1299 cells were seeded on sterile cover glasses placed in the 6-well plates. After overnight growth, cells were transfected with miR-941 mimics or NC sequence for 48 h. Briefly, a linear wound was created by dragging a 100-μl pipette tip through the monolayer before transfection. Cellular debris was removed by gentle washing with a culture medium, after which transfection was performed immediately. Low-serum (< 2%) culture medium was added, and the cells were allowed to migrate for a further 48 h. Subsequently, the dynamic wound-healing process was photographed using a microscope (Olympus 600 auto-biochemical analyzer; Olympus, Tokyo, Japan). The migration distance was measured from images (five fields) taken at each indicated time point. The residual gap between the migrating cells from the opposing wound edge was expressed as a percentage of the initial gap size.

### Annexin V-/PI apoptosis assay

In the investigation for miR-941 function, A549 and H1299 cells were seeded on sterile cover glasses placed in the 6-well plates. After overnight growth, cells were transfected with miR-941 mimics or NC sequence for 48 h. Pancreatic enzyme cell digestion solution (1 ml) without EDTA was added to the cell suspension, followed by centrifugation at 2,000 rpm for 5 min. The supernatant was then carefully aspirated. Subsequently, the cells were resuspended in binding buffer (250 ml), and the cell number was adjusted to 1 × 10^6^/ml. The cells were then incubated with Annexin V-FITC and propidium, washed with 1 × PBS, and fixed with 4% PFA for 10 min following the manufacturer’s instructions. Next, they were observed under BD FACSAria™ II flow cytometry, and the data were analyzed.

### Transwell assay

In this assay, Tumor cells were incubated in the upper chamber, while neutrophils were incubated in the lower chamber. In the investigation for miR-941 function, A549 and H1299 cells were seeded in upper chamber. After overnight growth, cells were transfected with miR-941 mimics or NC sequence for 48 h. After 48 h, the cells on the top surface of the membrane were mechanically removed using a cotton swab. Cells that had penetrated the membrane were stained with a crystal violet solution (4 g/L). The number of cells was counted under an inverted light microscope linked to a camera (Leica).

### Cell counting kit-8 assay

In the investigation for miR-941 function, A549 and H1299 cells were seeded in 96-well plates. After overnight growth, cells were transfected with miR-941 mimics or NC sequence for 48 h. LUAD cells were measured using cell proliferation reagent (WST-8; Roche, Germany). After plating cells in 96-well microtiter plates at 1.0 × 10^3^/well, 10 μl of CCK8 were added to each well at the time of harvest, according to the manufacturer’s instructions. Two hours after adding CCK8, cellular viability was determined by measuring the absorbance of the converted dye at 450 nm.

### Quantitative real-time PCR

Total RNA from cells or exosomes was extracted using TRIzol reagent (Invitrogen). The primer sequences for miR-941, FOXN4, U6 and GAPDH were designed and synthesized by GenePharma (China). RNAs were reverse‐transcribed with PrimeScript Reverse Transcriptase (Takara, Japan) or miRNA first strand cDNA synthesis (Sangon Biotech, China). The qPCR analysis was performed using SYBR Green detection kit (Takara, Japan) or a miRNA qPCR kit (Sangon Biotech, China) (SYBR Green method). All processes were performed according to the manufacturer’s instructions. GAPDH and U6 were used as endogenous controls. The primer sequences are listed in **Supplementary Table S1**.

### MiRNA and FOXN4 transfection

The miR-941 mimics were synthesized by the RiboBio Company (Guangzhou, China). LUAD cells were seeded in six-well plates 24 h before transfection. When the cells reached 50% confluency, the miRNA mimics were transfected with Lipofectamine 3000 according to the manufacturer’s instructions (Life Technologies). Briefly, 3 μl of Lipofectamine 3000 reagent was diluted in 100 μl of medium, and 5 μl (50 nM) of miR-941 mimics were diluted in 100 μl opti-mem (Gibco, Cat #31985062). Upon mixing the diluted reagents, the mixture was incubated at room temperature for 20 min and added to the cells. Forty-eight hours after transfection, the cells were harvested for later experiments. The FOXN4 coding sequence was obtained from Sangon Biotech Co. Ltd., Shanghai, China. The FOXN4 coding sequence was cloned into a pLVX-puro vector (Clontech, USA) to construct a lentiviral FOXN4- plasmid to overexpress FOXN4 in H1299 cells.

### Luciferase reporter assay

Amplified FOXN4 3’-UTR wild type (FOXN4 3’-UTR-WT) and corresponding FOXN4 3’-UTR-mutant (FOXN4 3’-UTR-MUT) were cloned into a pGL3 luciferase vector (Promega Corporation). H1299 cells were seeded in 96-well plates for 24 h. The cells were transfected with FOXN4 3’-UTR-WT or FOXN4 3’-UTR-MUT and miR-941 mimics using Lipofectamine 3000. After 48 h, the cells were harvested, and a Dual-Luciferase Reporter Assay Kit (Promega Corporation) was used to measure relative differences in luciferase activity.

### Nude mouse model

All the animal experiments in this study were approved by Affiliated Qingyuan Hospital of Guangzhou Medical University. A total of 12 four-week-old male BALB/c nude mice (GuangDong medical laboratory animal center, China) were randomized into two groups (*n* = 5 per group) and subcutaneously injected with 5 × 10^6^ miR-941 mimic-transfected H1299 cells (miR-941 mimic group) or vector H1299 cells (the control group). The tumor volume of each group was determined according to the following equation: tumor volume = 1/2 × (the largest diameter) × (the smallest diameter)^2^. The mice were euthanized by cervical dislocation after 22 days, and the volumes and wet weights of tumor tissues were determined every three days.

### Immunohistochemistry

The tumor tissue samples were fixed in 10% neutral formalin, embedded in paraffin, and cut into 4 μm thick sections. Slices underwent regular dewaxing and rehydration treatment, followed by thermally induced antigen repair. Endogenous peroxidase activity was blocked by 3% hydrogen peroxide, and nonspecific binding was blocked by 5% normal goat serum. Subsequently, the slices were incubated overnight with primary antibody at 4°C. The next day they were incubated with secondary antibody at room temperature for 1 h. The antibodies were as follows: Ki67 (Proteintech, Cat No: 27309-1-AP, 1:5000) and FOXN4 (Abcam, ab126757, 1:200). DAB staining, hematoxylin counterstaining, and finally gradient ethanol dehydration, paraffin sealing was conducted. Images were obtained using an Olympus BX53 microscope.

### Western blot

H1299 cells were inoculated into a six-well plate at a density of 2 × 10^6^ cells per well for 24 h. After transfection with FOXN4 for 24 h, the cells were collected to extract total cell proteins. The protein (20 μg) was loaded and separated by polyacrylamide gel electrophoresis. After electrophoresis, the protein was transferred to a PVDF membrane. FOXN4 (Abcam, ab126757, 1:20000), N-cadherin (Proteintech, 22018-1-AP, 1:3000), E-cadherin (Proteintech, 20874-1-AP, 1:5000), Vimentin (Proteintech, 80232-1-RR, 1:50000), p-smad2 (Abcam, ab300079, 1:1000), Smad2 (Abcam, ab40855, 1:5000), TGF-β2 (Proteintech, 21898-1-AP, 1:2500), and GAPDH (Proteintech, 60004-1-Ig, 1:10000) were incubated overnight.

### Statistical analysis

All statistical analyses were performed using GraphPad Prism 10.0 (GraphPad Software, San Diego, CA, USA). Differences were examined using the student’s t-test or one-way analysis of variance. The data are presented as means ± SD. A value of *p* < 0.05 was considered statistically significant. Survival outcomes were analyzed using Kaplan-Meier curves and log-rank tests. Multivariate Cox regression models were constructed to adjust for potential confounders, including demographic variables (age, gender), clinical parameters (TNM stage, smoking history), and treatment modalities (chemotherapy/radiotherapy).

## Results

### The miRNA activity was significantly changed in TANs of LUAD based on scRNA-seq analysis

**Figure 1:**
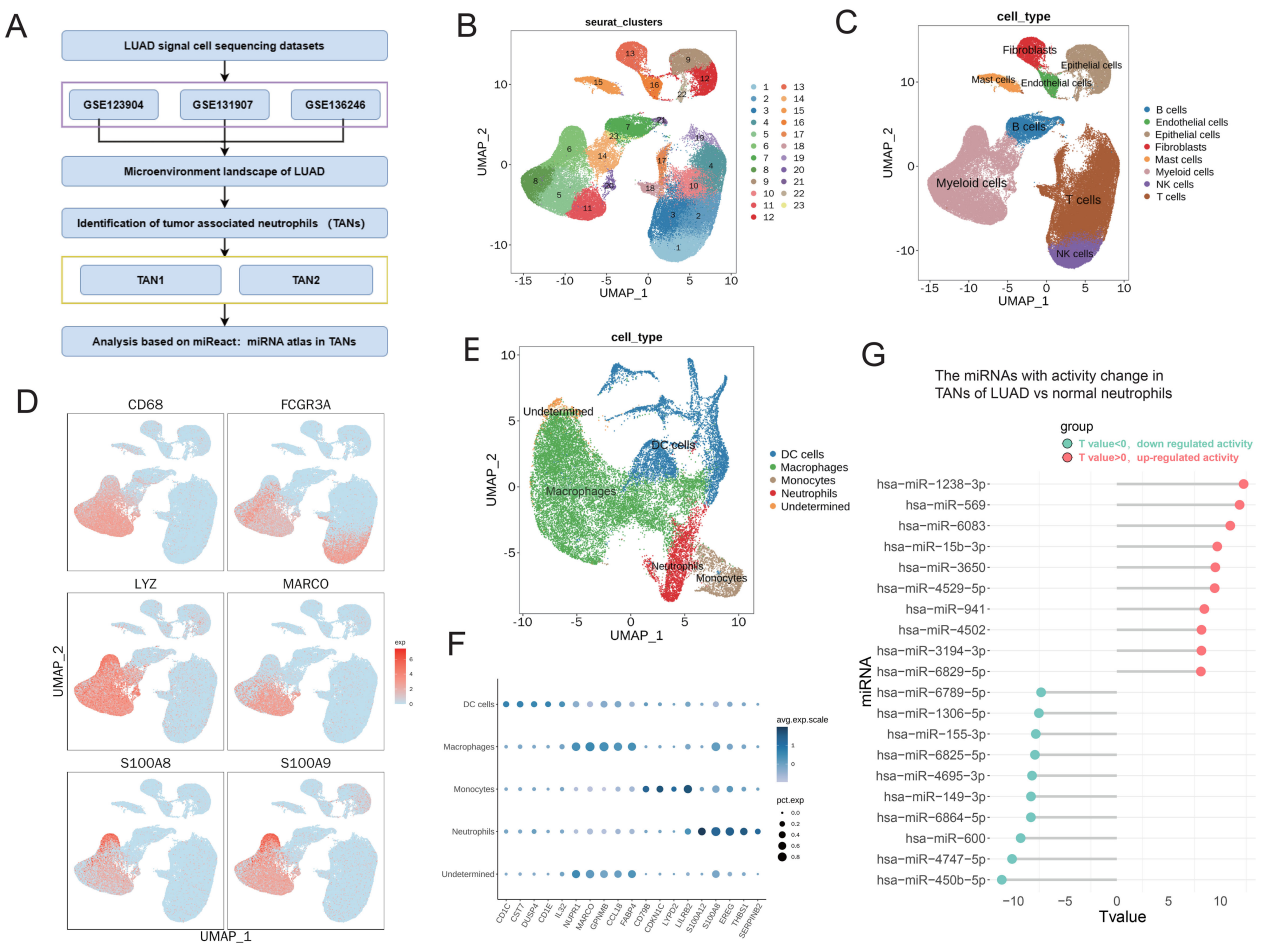
The miRNA activity was significantly changed in TANs of LUAD based on scRNA-seq analysis. **(A)** Workflow of bioinformatics analysis based on sc-RNA datasets of LUAD (GSE123902, GSE131907, and GSE136246). **(B, C)** UMAP plots showing the identified clusters **(B)** and cell lineages **(C)** in LUAD. **(D)** Specific markers of myeloid cells are shown in UMAP plots. **(E)** UMAP plots showing the identified cell lineages of myeloid cells, including TANs, in LUAD. **(F)** Bubble chart displaying characteristic biomarkers of myeloid cell lineages. **(G)** Lollipop chart displaying top 10 miRNAs with significantly altered activity in neutrophils. The activity of miRNAs was calculated using the miReact algorithm.

First, bioinformatics analysis based on scRNA-seq datasets of LUAD (GSE123902, GSE131907, and GSE136246) was performed to reveal the microenvironment landscape of LUAD (**Figure 1A**). There were 56,142 cells in the LUAD samples and 45,575 cells in the normal samples. As shown in **Figures 1B, C**, 24 clusters and eight cell types were obtained through dimensionality reduction clustering. Among these eight cell types, myeloid cells were selected for further analysis. The identification markers for myeloid cells features included CD68, FCGR3A, LYZ, MARCO, S100A8, and S100A9 (**Figure 1D**). Subsequently, myeloid cells were identified, including DC cells, macrophages, monocytes, and neutrophils (**Figures 1E, F**). Neutrophils in the TME (i.e., TANs) are closely related to multiple aspects of tumor, such as the occurrence, development, invasion, and metastasis of tumors. To determine miRNA activity from neutrophils in TME, we used the miReact algorithm. The results revealed a significant increase in the activity of miRNAs, including has-miR-569, has-miR-15b-3p, and hsa-miR-941 (**Figure 1G**). These results suggest that neutrophil-derived miRNA in TME may play a key role in regulating the progression of LUAD.

### The miRNA activity was significantly changed between TANs subtypes in LUAD

**Figure 2:**
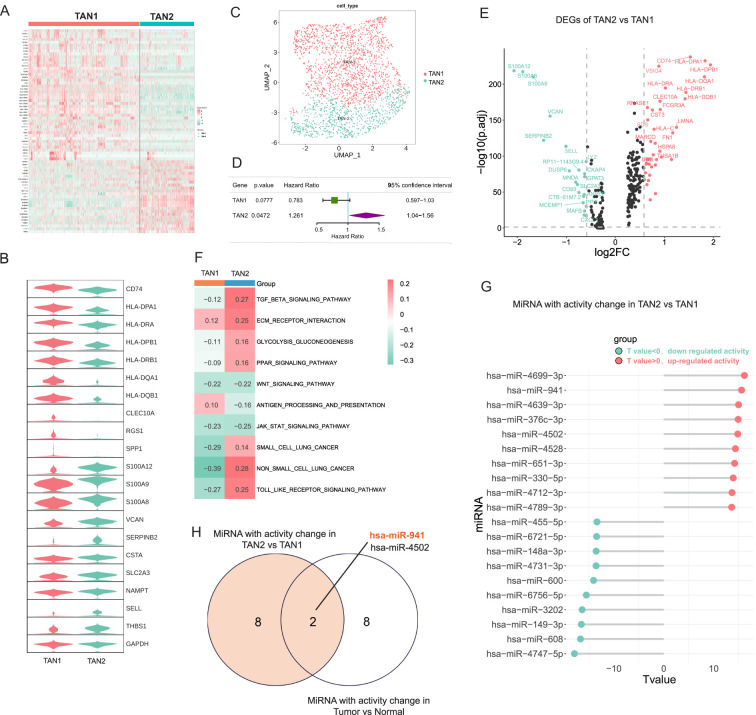
The miRNA activity was significantly changed between TANs subtypes in LUAD. **(A)** Heatmap displaying the characteristic markers of TAN subsets of TAN1 and TAN2. **(B)** Violin chart showing significant changes in the characteristic markers of TAN1 and TAN2. **(C)** UMAP plots showing TAN subsets of TAN1 and TAN2. **(D)** The Prognostic effects of TAN1 and TAN2 signatures are evaluated by multivariate Cox regression. **(E)** DEGs between the TAN1 and TAN2 subsets. **(F)** GSVA analysis comparing pathway activity among TAN1 and TAN2 subtypes by enrichment scores. **(G)** Lollipop chart displaying the top 10 miRNAs with significantly altered activity in TAN2 versus TAN1. **(H)** The Venn diagram shown the common top 10 miRNAs with activity change in Tumor *vs* normal and TAN2 *vs* TAN1 group.

TANs exhibit heterogeneity and can be classified into different subgroups, with the N1 type typically associated with anti-tumor activity and the N2 type associated with protumor activity (49). To reveal the heterogeneity of TANs in LUAD, two major subtypes of TANs (TAN1 and TAN2) were classified based on their distinctive transcriptomic profiles in LUAD. There were 1,303 cells in the TAN1 subtype and 999 cells in the TAN2 subtype. TAN1 highly expressed antigen presentation-related genes, such as HLA–DPB1. The TAN2 subtype exhibited strong expression of canonical markers of TANs, including S100A8/9 (**Figures 2A–C**). The prognostic effects of the TAN2 signature were evaluated using multivariate Cox regression analysis, adjusting for age, gender, TNM stage, and smoking status (Hazard Ratio (HR): 1.261, p = 0.0472, 95% confidence interval: 1.044–1.56) (**Figure 2D**), indicating the clinical relevance of the TAN2 signature. To reveal the molecular mechanisms of the TAN subtypes, DEGs between TAN1 and TAN2 were identified (**Figure 2E**). GSVA was performed to assess the variation of pathway activity between the two TAN subtypes (**Figure 2F**). Significant upregulation of the TGF-β signaling pathway was observed, in addition to enhanced pathway activity associated with lung cancer process. TAN1 genes were significantly enriched in antigen-processing signaling pathways. These results suggest that TAN2 is closely related to N2 neutrophils. **Figure 2G** shows miRNAs with significantly altered activity in TAN2 versus TAN1, together with high activity of some miRNAs, such as has-miR-941, has-miR-330-5p, and has-miR-651-3p. To identify miRNAs that play an important role and exhibit activity changes in the TAN of LUAD, we intersected the activity changes of miRNAs in Tumor *vs* normal and TAN2 *vs* TAN1, and found that miR-941 had the most significant common activity changes within them (**Figure 2H** **).** Hence, the miR-941 was chosen for the following experiment.

### MiR-941 serves as an oncogenic miRNA in LUAD

**Figure 3:**
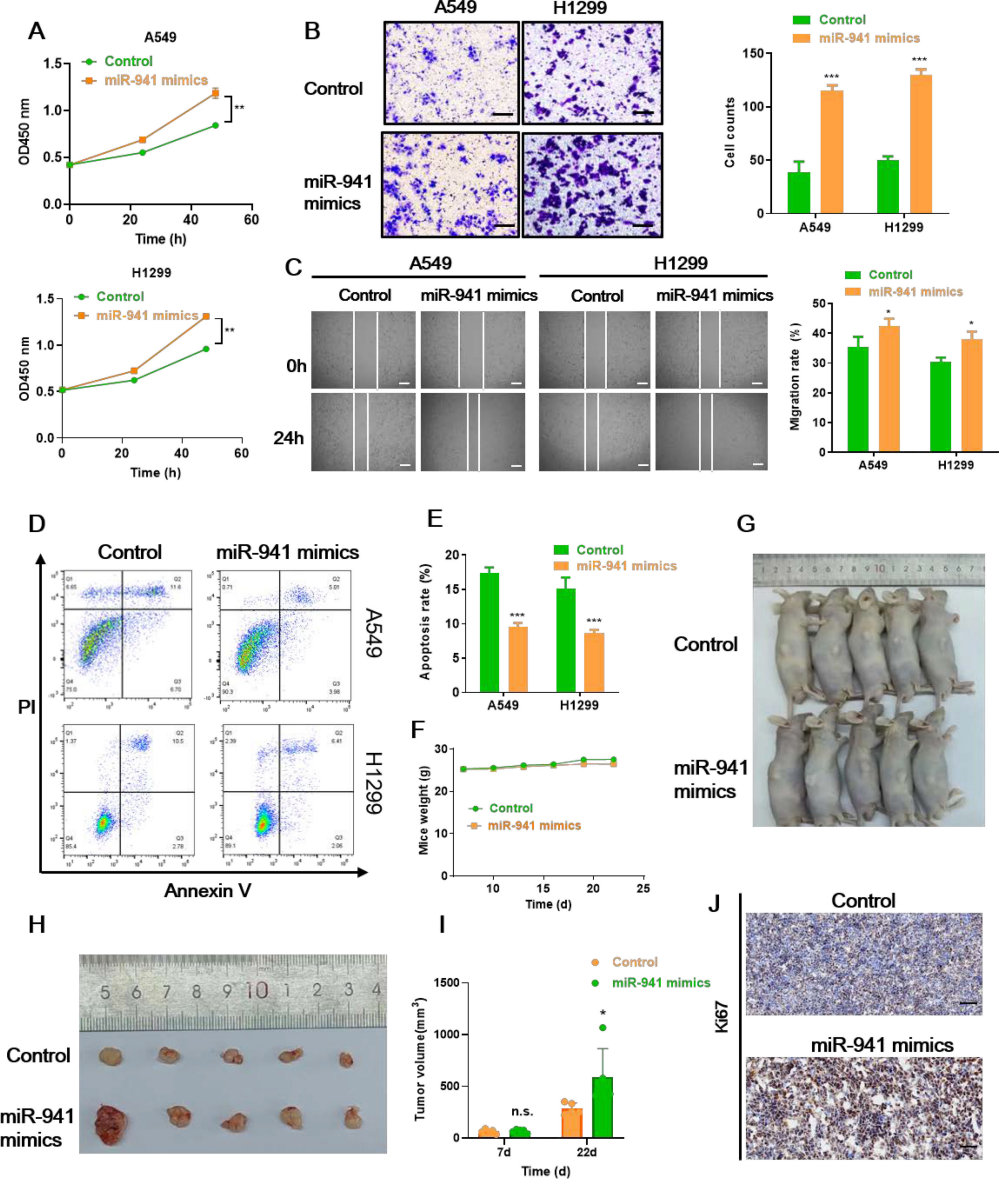
The miR-941 mimic facilitates LUAD tumor growth *in vitro* and *in vivo*. **(A–C)** CCK8 assays **(A)**, transwell assays **(B)**, and wound healing assays **(C)** showing the proliferation, invasion, and migration capability of A549 and H1299 lung cancer cells following the treatment with miR-941 mimics. Scale bar: 50 μm for transwell assays, 200 μm for scratch assays. **(D, E)** Annexin V/PI staining assays showing the apoptosis rate of A549 and H1299 lung cancer cells following the treatment with miR-941 mimics. **(F)** Weights of the mice in the control and miR-941 mimic groups. **(G)** Representative image of nude mice in the control and miR-941 mimic groups. **(H)** Representative image of tumors collected from nude mice in the control and miR-941 mimic groups. **(I)** Volume of mice in the control and miR-941 mimic groups. **(J)** Immunohistochemical staining showing the expression of Ki67 in the control and miR-941 mimic groups. Scale bar: 50 μM. *N* = 5, n.s. indicates not significantly, *p < 0.05, **p < 0.01; ***p < 0.001 *versus* the control group.

To uncover the function of miR-941 in LUAD cell lines, we transfected A549 and H1299 cells with miR-941 mimics and investigated their impact on cell proliferation, apoptosis, migration, and invasion. As shown in **Figure 3A**, miR-941 mimics transfection significantly increased the number of A549 and H1299 cells, respectively. The transwell and wound healing assay results showed that A549 and H1299 cells transfected with miR-941 mimics exhibited significantly higher invasion and migration capabilities (**Figures 3B, C**). In addition, the apoptosis rates of both cell lines were significantly decreased in the miR-941 mimics group, with 12.92% *versus* 5.91% (control *vs*. miR-941 mimics) and 14.91% *versus* 6.08% (control *vs*. miR-941 mimics) in A549 and H1299 cells, respectively (**Figures 3D, E**). Moreover, miR-941 in the nude mouse xenograft model promoted mice tumor growth compared with the control group (**Figures 3F–I**). The IHC of Ki67 (tumor cell proliferation marker) on mice tumor tissue suggests that the miR-941 mimic promotes the proliferation of lung cancer cells (**Figure 3J**). Taken together, these results prove that miR-941 treatment promoted malignant phenotype of LUAD cells, while how miR-941 functions remain to be explored.

### Prediction targets of miR-941 and prognostic significance of FOXN4 in LUAD

**Figure 4:**
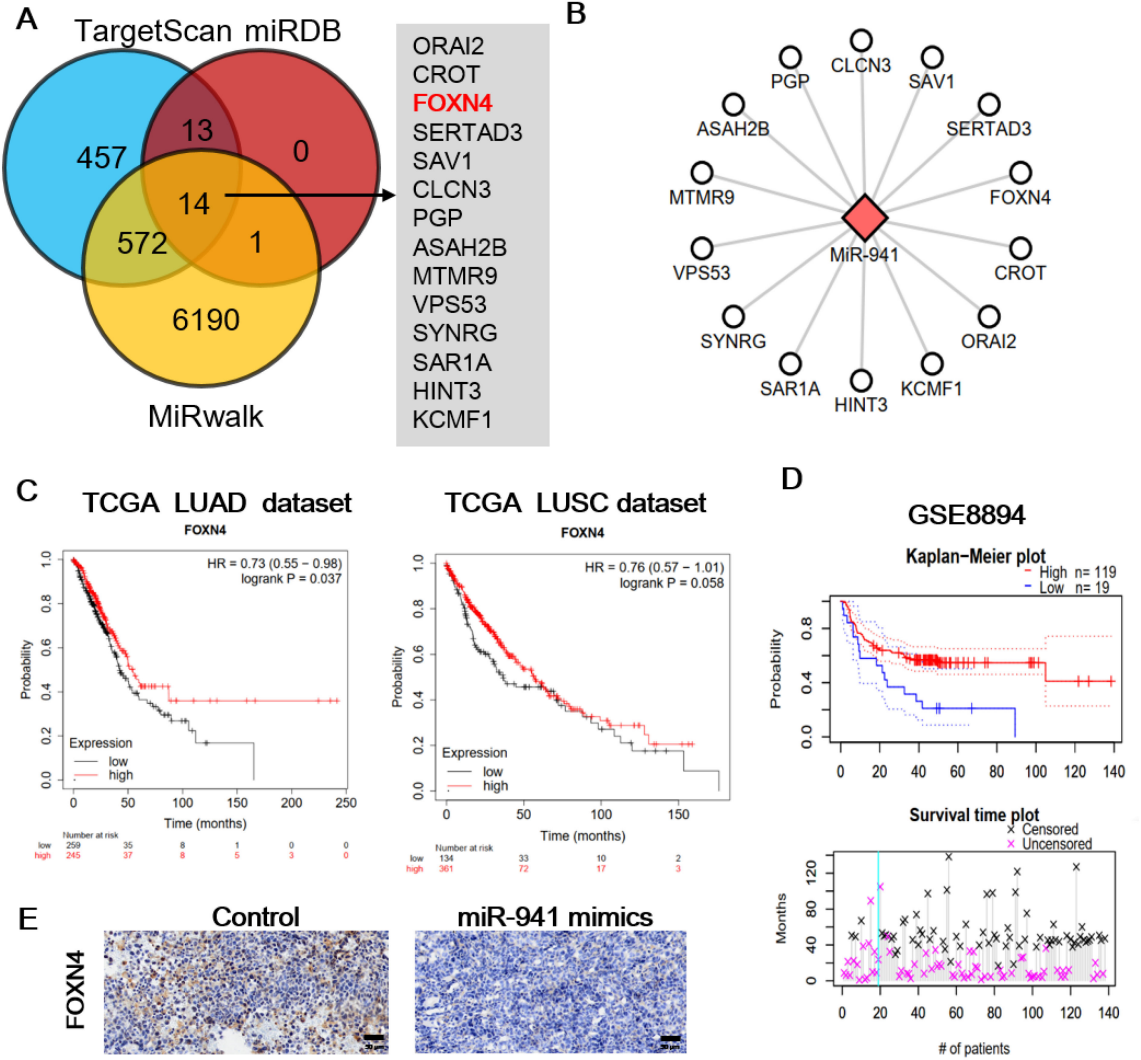
Prediction targets of miR-941 and prognostic significance of FOXN4 in LUAD. **(A)** miR-941 targets were predicted using TargetScan, miRDB, and MiRwalk, and common targets were identified. **(B)** Network showing the relationship between miR-941 and common targets. **(C)** Prognostic significance of FOXN4 in TCGA, LUAD, and LUSC datasets. **(D)** Prognostic significance of FOXN4 in the GSE8894 lung cancer dataset. **(E)** Immunohistochemical staining showing the expression of FOXN4 in the control and miR-941 mimic groups. .

To explore the downstream mechanism of miR-941, we first predicted potential targets with three database and online tools: TargetScan, miRDB, and MiRwalk (**Figure 4A**). Fourteen genes were predicted using all three tools: ORAI2, CROT, FOXN4, SERTAD3, SAV1, CLCN3, PGP, ASAH2B, MTMR9, VPS53, SYNRG, SAR1A, HINT3, and KCMF1 (**Figure 4B**). We then investigated the functions and clinical significance of these genes in LUAD. Only forkhead box N4 (FOXN4) showed significant protective effect on survival in LUAD patients. As shown in **Figure 4C** and **Figure 4D**, hazard ratios of FOXN4 were as follow: TCGA LUAD (0.73), lung squamous carcinoma (LUSC) (0.76) and the LUAD dataset of GSE8894 (0.44). The IHC of FOXN4 on mice tumor tissue presented the lower expression of FOXN4 with the treatment of the miR-941 mimic (**Figure 4E**).

**Figure 5:**
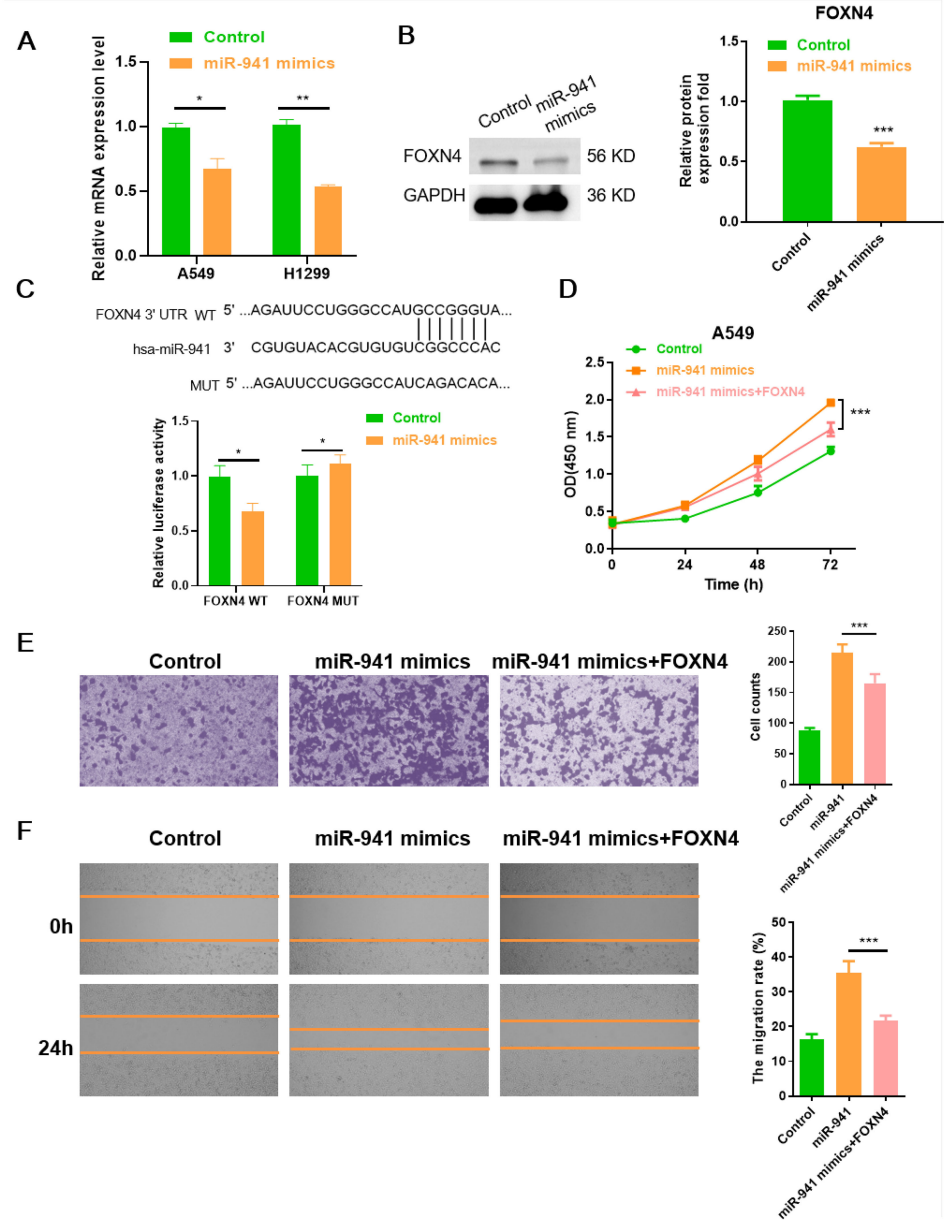
The miR-941-FOXN4 axis contributes to the malignant phenotype of LUAD cells. **(A)** Relative quantification of mRNA level of FOXN4 in miR-941 mimic-treated A549 and H1299 cell lines. **(B)** Relative protein expression level of FOXN4 in miR-941 mimic-treated A549 cell line. **(C)** Luciferase assays showing the regulation of miR-941 on FOXN4. **(D)** CCK8 assays showing proliferation of LUAD A549 cells following treatment with miR-941 mimics or miR-941+FOXN4. **(E)** Transwell assays showing invasion of LUAD A549 cells following treatment with miR-941 mimics or miR-941+FOXN4. Scale bar: 50 μm. **(F)** Wound healing assays showing the migration of A549 cells after treatment with miR-941 mimics or miR-941+FOXN4. Scale bar: 200 μm. *N* = 3, *p < 0.05; **p < 0.01, ***p < 0.001.

Next, to further verify FOXN4 as the target of miR-941 in the LUAD cell lines, FOXN4 mRNA expression was examined with RT-qPCR in miR-941 mimics treated A549 and H1299 cells, respectively. As shown in **Figure 5A**, FOXN4 mRNA expression was significantly decreased in the miR-941 mimic-treated cell lines. As presented in **Figure 5B**, the protein expression level of FOXN4 was also remarkably reduced in the miR-941-treated cells. We conducted a luciferase assay to explore the direct binding sites of FOXN4 3’-UTRs with miR-941. The results showed that the miR-941 mimics significantly reduced luciferase intensity in the FOXN4 3’-UTR-WT group, whereas no significant change was observed in the FOXN4 3’-UTR-MUT group (**Figure 5C**), supporting the conclusion that miR-941 inhibits FOXN4 in LUAD. Furthermore, the effect of miR-941 on promoting the malignant phenotype (proliferation, invasion, and migration) of LUAD cells was rescued by overexpressing FOXN4 (**Figures 5D–F**).

### TANs promoted LUAD progression via a miR-941/FOXN4/TGF-β feedback signaling loop

**Figure 6:**
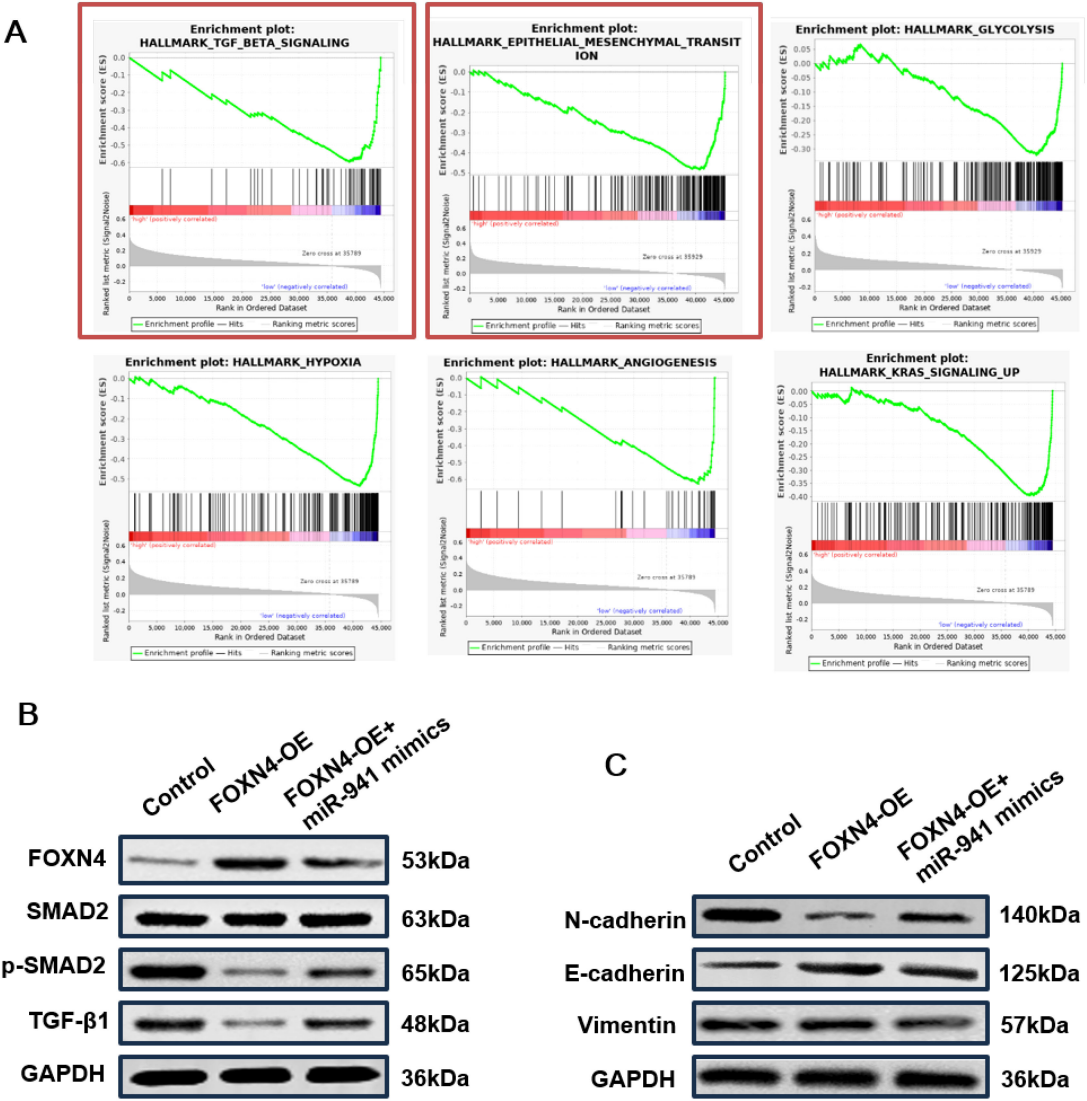
TANs promote LUAD progression via a miR-941/FOXN4/TGF-β feedback signaling loop **(A)** GSEA hallmark pathways in lung cancer patients with high expression of FOXN4, including the TGF-β signaling pathway and EMT signaling pathway. **(B)** WB blot showing the relative expression levels of key proteins in the TGF-β signaling pathway of the H1299 cell line treated with overexpressed FOXN4 (FOXN4-OE) and FOXN4-OE + miR-941 mimic. **(C)** WB showing the relative expression levels of key proteins in the EMT signaling pathway of the H1299 cell line treated with overexpressed FOXN4 (FOXN4-OE) and FOXN4-OE + miR-941 mimic. *N* = 3.

As FOXN4 has been reported to inhibit cancer progression, downstream pathways in LUAD were analyzed using the TCGA datasets. Based on the expression of FOXN4 in the LUAD and LUSC datasets, we divided LUAD samples into FOXN4 high and low groups and used GSEA to enrich significant hallmark pathways in the FOXN4 group. As shown in **Figure 6A**, multiple tumor progression-inducing pathways were deactivated in the FOXN4 high group including the TGF-β signaling pathway and hypoxia, epithelial-mesenchymal transition (EMT), glycolysis, angiogenesis, and K-RAS signaling pathways, implying that FOXN4 exerts a tumor-suppressing function in LUAD by repressing these pathways. The inhibitory role of FOXN4 in the TGF-β signaling pathway and EMT process was verified by WB. Overexpression of FOXN4 resulted in a marked decrease in the expression of key proteins, such as TGF-β1 and p-smad2/smad2 (**Figure 6B**), in the TGF-β signaling pathway. N-cadherin expression, a biomarker of EMT, was also markedly decreased, whereas E-cadherin and vimentin expression were significantly enhanced (**Figure 6C**).

**Figure 7:**
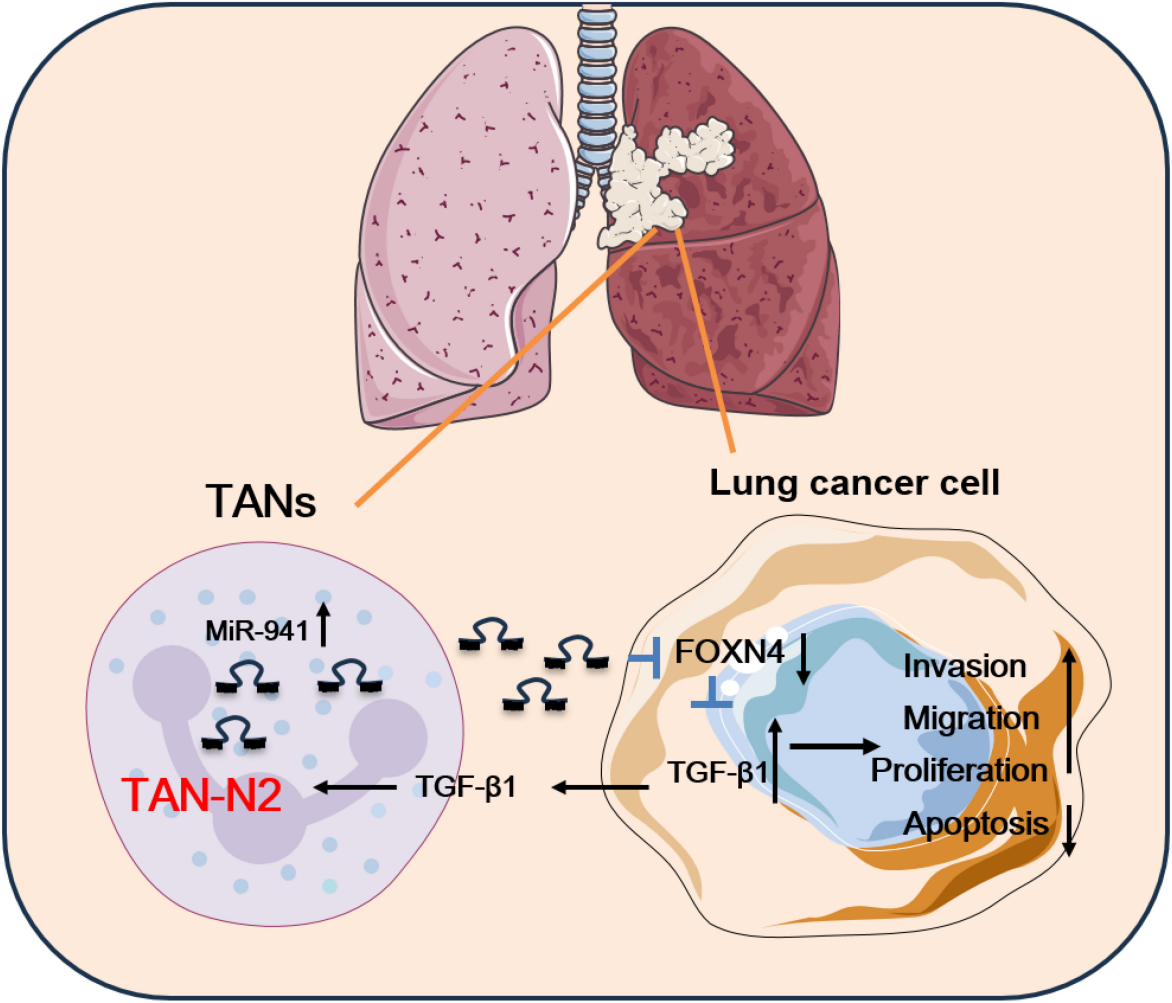
The schematic diagram of this study. In this study, miR-941 served as a TAN-secreted oncogenic gene in LUAD by decreasing FOXN4 expression and activating the TGF-β signaling pathway. The miR-941 upregulated the expression of TGF-β1, which is secreted by LUAD cells. This, in turn, stimulated TAN polarization into the TAN2 subtype. TANs in LUAD promoted the malignant behavior of lung cancer cells via a miR-941/FOXN4/TGF-β feedback loop.

Collectively, the results support the conclusion that N2 TANs secret upregulation of miR-941 in the TME as a cross-talk mediator between TANs and lung cancer cells. The miR-941 promoted LUAD progression via a FOXN4/TGF-β feedback signaling loop. TGF-β in the feedback loop induced N2 polarization of TANs (**Figure 7**).

## Discussion

In LUAD, the intricate interplay between immune cells has recently attracted much attention, offering a promising avenue for understanding disease progression and predicting immunotherapy responses (15, 50–53). Notably, as a population of inflammation-related myeloid cells (54, 55), TANs were reported to promote tumor cell survival and extravasation through integrin mediation (56). Clinically, neutrophil-related parameters, like the neutrophil-to-lymphocyte ratio, can be used as markers for immune checkpoint inhibitor treatment and prognosis prediction in lung cancer (57–61). Functionally, both pro- and antitumor roles of neutrophils have been reported in LUAD progression (35, 62–66), and cytokines and receptors (e.g., CXCR2) are pivotal mediators in this process (67). Accumulation of CCR1+TANs was reported in lung metastasis of colorectal cancer CCL15-CCR1 axis (68). Studies have also shown that neutrophils promote lung metastasis in breast cancer by serving as an energy reservoir (69) in a leukotriene-generating enzyme arachidonate 5-lipoxygenase-dependent way (70). Singhal et al. demonstrated the existence of a specialized subset of TANs with anti-tumor capabilities in early-stage human LUAD (71). Targeting TANs, such as CXCR2 (72) and cathepsin C (73), has recently become a novel immunotherapeutic strategy in LUAD (74). Therefore, elucidating functional genes and pathways of TANs in LUAD would provide a fundamental basis for both pathogenesis and clinical application.

In our study, TANs in LUAD were divided into TAN1 and TAN2 cell subtypes based on a scRNA-seq dataset. The TAN2 cell subtype highly expressed a biomarker of N2 neutrophils. The N2 type has been reported to be associated with protumor activity (75). N2 neutrophils can promote tumor metastasis by expressing arginase, matrix metalloproteinase-9 (MMP-9), vascular endothelial growth factor, and various chemokines,(including CCL2, CCL5, and CXCL4 (76). N1 and N2 neutrophils can transform into each other. Various factors affect the polarization of neutrophil phenotypes. Among these, N1 neutrophils can be transformed into the N2 phenotype via the mediation of TGF-β (77). Of note, the TGF-β signaling pathways can regulate the phenotypic transition of neutrophils. Interestingly, in our study, when co-cultured with TANs, more TGF-β1 was secreted by lung cancer cells in the supernatant and induced N2 polarization of TANs.

The miRNAs are small noncoding RNAs that play a post-transcriptional regulatory role in diseases. In LUAD, studies have concentrated on cancer cells and revealed both pro- and anti-tumor functions of miRNAs and their clinical significance (78, 79). For instance, studies have uncovered the function of miR-21 in tumorigenesis (80); miR-9, miR-708, and miR-126 in metastasis (81–83); and miR-143/145 in neoangiogenesis (84). Clinically, individual miRNAs, such as miR-205 (85) have been shown to perform well as diagnostic and prognostic biomarkers (86–90). Recently, the role of miRNAs in immune cells and cell–cell communication in the TME and LUAD progression has attracted attention (16). Fortunato et al. reported that circulating miR-320a promotes an immunosuppressive macrophage M2 phenotype through the downregulation of STAT4, increasing the risk of LUAD (91). Lv et al. showed that decreased expression of miR-141 resulted in increased production of CXCL1 and recruitment of Tregs to promote immune escape of the tumor (92). By promoting tumor-infiltrating lymphocyte infiltration, miR-15 was shown to function as a novel immune system activator (93). For neutrophils in cancer, miR-338-3p and miR-199b-5p were found to be associated with the absolute neutrophil count in patients with resectable pancreatic cancer (94). In hepatocellular carcinoma, miR-301b-3p showed a positive correlation with TANs and played a central role in the interaction between cancer stem-like cells and TANs (95). In other research, miR-125a was shown to be a critical modulator of neutrophil development (96).

In this study, we provided a new reference for understanding the function of miR-941 secreted by TANs in LUAD. In laryngeal squamous cell carcinoma, serum exosomal miR-941 was shown to be an oncogenic biomarker and potential therapeutic target (97). In hepatoma, miR-941 regulated the EMT process and affected cell migratory/invasive properties (98). In breast cancer, an miR-941 inhibitor significantly decreased the proliferation and migration of MDA-MB-231 cells by altering the expression of onco-proteins, including p21, cyclin D1, PP2B-B1, and MMP-13 (99). The diagnostic potential of miR-941 in colon cancer and hepatocellular carcinoma has been demonstrated (100, 101). Our is the first study to reveal the function of miR-941 in LUAD and link miR-941 with the function of TANs in tumorigenesis. This study sheds light on the significance of miR-941 in LUAD, emphasizing its potential as a therapeutic target.

The present study demonstrates that FOXN4 is a target of miR-941. The results of a combination of experimental validation, expression analyses, and luciferase assays establish FOXN4 as a critical downstream effector of miR-941. A previous study reported the potential role of FOXN4 in the immune response in LUAD and its prognostic significance (102), which is consistent with the results of this study. In addition, the GSEA analysis in this study showed the depression of multiple cancer-promoting pathways, such as the TGF-β signaling pathway, hypoxia, EMT, glycolysis, AND angiogenesis, as well as K-RAS signaling pathways, implying that FOXN4 exerts a tumor-suppressive effect in LUAD by repressing these pathways. During the process of the TGF-β pathway-induced EMT, the expression of N-cadherin was reported to be upregulated (103). In the EMT process, epithelial cells gain N-cadherin mediated adhesion properties, which help them acquire mesenchymal cell characteristics, such as enhanced motility and invasiveness (104). Our WB results indicated that FOXN4 overexpression reduced the protein expression of N-cadherin, implying that miR-941 targeted FOXN4 to promote the EMT process. Interestingly, previous studies have indicated that TGF-β induces the tumor-associated neutrophil to N2 phenotype (105). MiRNAs has also been reported to be involved in the signaling axis of tumor associated neutrophil N2 polarization induced by TGF-β. For instance, Shang et al. have indicated that exosomal circPACRGL promotes progression of colorectal cancer via the miR-142-3p/miR-506-3p- TGF-β1 axis (77). Hence, we speculated that the miR-941/FOXN4/TGF-β axis induces N2 polarization of neutrophils and enhances tumor progression of LUAD.

The clinical relevance of miR-941 as a therapeutic target in LUAD is multifaceted. While our study demonstrates that miR-941 mimics exacerbate tumor progression *in vitro* and *in vivo*, therapeutically, targeting miR-941 suppression (rather than mimicking its oncogenic activity) would be the logical strategy. Potential clinical applications include antisense oligonucleotides (ASOs) or miRNA inhibitors, such as locked nucleic acid (LNA)-based anti-miR-941 agents, to neutralize its oncogenic effects. For instance, systemic delivery of miR-941 inhibitors via lipid nanoparticles could suppress its activity in TANs and tumor cells, thereby disrupting the miR-941/FOXN4/TGF-β feedback loop. Additionally, blocking miR-941 transfer from TANs to tumor cells could be achieved through exosome-mediated strategies, such as using exosomal inhibitors (e.g., GW4869) or engineering exosomes to deliver FOXN4 mRNA or TGF-β pathway antagonists. Furthermore, combining miR-941 inhibition with TGF-β pathway inhibitors (e.g., galunisertib) or immune checkpoint blockade (e.g., anti-PD-1/PD-L1) may enhance antitumor immunity and reverse N2-TAN polarization. Finally, circulating miR-941 levels in plasma or neutrophil-derived exosomes could serve as a non-invasive biomarker to predict immunotherapy response or monitor disease progression.

While this study elucidates the critical role of the miR-941/FOXN4/TGF-β signaling axis in LUAD progression and TAN polarization, several limitations should be acknowledged. First, although TAN1 and TAN2 subsets were identified via scRNA-seq, their functional heterogeneity—such as the clinical validation of N1/N2-specific surface markers or cytokine profiles—remains incompletely characterized. Second, while miR-941 overexpression drives LUAD malignancy *in vitro* and *in vivo*, direct evidence of miR-941 secretion from TANs and its transfer to tumor cells via exosomes or extracellular vesicles requires further experimental validation. Third, our focus on FOXN4 as a primary miR-941 target does not exclude contributions from additional downstream effectors or parallel pathways.

To address these gaps, future investigations should prioritize validating N1/N2 TAN markers (e.g., CXCR2, PD-L1) in clinical cohorts using spatial transcriptomics to map miR-941’s spatial regulation within the tumor-stroma-neutrophil niche. Building on this, elucidating miR-941 delivery mechanisms—for instance, by blocking exosomal transfer through GW4869 treatment or Rab27a knockdown—will clarify its role in intercellular communication. Furthermore, developing therapeutic strategies such as miR-941-targeted antisense oligonucleotides or nanoparticle-based delivery systems, particularly when combined with TGF-β inhibitors (e.g., galunisertib) or immune checkpoint blockade, could disrupt the miR-941/FOXN4/TGF-β feedback loop. Finally, translating miR-941 into clinical practice by correlating its levels in plasma or neutrophil-derived exosomes with patient responses to immunotherapy or TGF-β-targeted therapies in trials may establish its utility as a liquid biopsy biomarker. Collectively, these efforts will advance our understanding of miR-941’s regulatory network and provide novel therapeutic avenues for reprogramming immunosuppressive TANs in LUAD.

Collectively, the results support the conclusion that N2 TANs secrete upregulation of miR-941 in the TME as the cross-talk mediator between TANs and lung cancer cells. By participating in a FOXN4/TGF-β feedback signaling loop, miR-941 plays an oncogenic role in the process of TANs enhancing cancer cell malignant behavior. TGF-β in the feedback loop induces N2 polarization of TANs. This study sheds light on their collaborative impact on LUAD progression and offers avenues for future therapeutic interventions.

## Data Availability

Publicly available datasets were analyzed in this study. This data can be found here: LUAD scRNA-seq datasets (GSE123902, GSE131907, and GSE136246) were obtained from the GEO database.

## Supplementary material

Supplementary Table 1 The primer sequences used in this study.
