# Supplementary material for: The miR-941/FOXN4/TGF-β feedback loop induces N2 polarization of neutrophils and enhances tumor progression of lung adenocarcinoma
Source: Front Immunol. 2025 Apr 25;16:1561081. doi: 10.3389/fimmu.2025.1561081 (PMC12061992; doi:10.3389/fimmu.2025.1561081)
Supplement: Supplementary Table 1 — The primer sequences used in this study. [file Table1.docx]

**Supplemental Table**

**Table S1. The primer sequences used in this study.**

| Gene | Forward, 5ʹ-3 ʹ | Reverse, 5ʹ-3 ʹ |
| --- | --- | --- |
| CXCR2 | CCTGTCTTACTTTTCCGAAGGAC | TTGCTGTATTGTTGCCCATGT |
| miR-941 | GCACCCGGCTGTGT | CTCAACTGGTGTCGTGGA |
| FOXN4 | AGGGCTCCTGTAGACTTCATC | CCAAGCTGAATCCCTCATCCT |
| U6 | AAAGCAAATCATCGGACGACC | GTACAACACATTGTTTCCTCGGA |
| GAPDH | TGTGGGCATCAATGGATTTGG | ACACCATGTATTCCGGGTCAAT |
